# Quantitative evaluation of hepatic integrin α_v_β_3_ expression by positron emission tomography imaging using ^18^F-FPP-RGD_2_ in rats with non-alcoholic steatohepatitis

**DOI:** 10.1186/s13550-020-00704-3

**Published:** 2020-10-07

**Authors:** Shuichi Hiroyama, Takemi Rokugawa, Miwa Ito, Hitoshi Iimori, Ippei Morita, Hiroki Maeda, Kae Fujisawa, Keiko Matsunaga, Eku Shimosegawa, Kohji Abe

**Affiliations:** 1 Translational Research Unit, Biomarker R&D Department, Shionogi & Co., Ltd., 3-1-1 Futaba-cho, Toyonaka, Osaka 561-0825 Japan; 2 Research Laboratory for Development, Shionogi & Co., Ltd., 3-1-1 Futaba-cho, Toyonaka, Osaka 561-0825 Japan; 3 Laboratory for Advanced Medicine Research, Shionogi & Co., Ltd., 3-1-1 Futaba-cho, Toyonaka, Osaka 561-0825 Japan; 4 Laboratory for Innovative Therapy Research, Shionogi & Co., Ltd., 3-1-1 Futaba-cho, Toyonaka, Osaka 561-0825 Japan; 5 Department of Molecular Imaging in Medicine, Graduate School of Medicine, Osaka University, 2-2 Yamadaoka, Suita, Osaka 565-0871 Japan

**Keywords:** Non-alcoholic fatty liver disease (NAFLD), Non-alcoholic steatohepatitis (NASH), Fibrosis, Integrin, Arginine–glycine–aspartic acid (RGD), Positron emission tomography (PET)

## Abstract

**Background:**

Integrin α_v_β_3_, which are expressed by activated hepatic stellate cells in non-alcoholic steatohepatitis (NASH), play an important role in the fibrosis. Recently, we reported that an RGD peptide positron emission tomography (PET) probe is useful as a predictor of hepatic fibrosis. Kinetic analysis of the RGD PET probe has been performed in tumours, but not in hepatic fibrosis. Therefore, we aimed to quantify hepatic integrin α_v_β_3_ in a model of NASH by kinetic analysis using ^18^F-FPP-RGD_2_, an integrin α_v_β_3_ PET probe.

**Methods:**

^18^F-FPP-RGD_2_ PET/CT scans were performed in control and NASH rats. Tissue kinetic analyses were performed using a one-tissue, two-compartment (1T2C) and a two-tissue, three-compartment (2T3C) model using an image-derived input function (IDIF) for the left ventricle. We then conducted correlation analysis between standard uptake values (SUVs) or volume of distribution (*V*_T_), evaluated using compartment kinetic analysis and integrin α_v_ or β_3_ protein expression.

**Results:**

Biochemical and histological evaluation confirmed the development of NASH rats. Integrin α_v_β_3_ protein expression and hepatic SUV were higher in NASH- than normal rats. The hepatic activity of ^18^F-FPP-RGD_2_ peaked rapidly after administration and then gradually decreased, whereas left ventricular activity rapidly disappeared. The 2T3C model was found to be preferable for ^18^F-FPP-RGD_2_ kinetic analysis in the liver. The *V*_T (IDIF)_ for ^18^F-FPP-RGD_2_, calculated using the 2T3C model, was significantly higher in NASH- than normal rats and correlated strongly with hepatic integrin α_v_ and β_3_ protein expression. The strengths of these correlations were similar to those between SUV_60–90 min_ and hepatic integrin α_v_ or β_3_ protein expression.

**Conclusions:**

We have demonstrated that the *V*_T (IDIF)_ of ^18^F-FPP-RGD_2_, calculated using kinetic modelling, positively correlates with integrin α_v_ and β_3_ protein in the liver of NASH rats. These findings suggest that hepatic *V*_T (IDIF)_ provides a quantitative assessment of integrin α_v_β_3_ protein in liver.

## Background

Non-alcoholic fatty liver disease (NAFLD) is one of the most common causes of chronic liver disease [1, 2], and the more severe form of this condition, non-alcoholic steatohepatitis (NASH), is a serious disease [3]. Approximately half of NASH patients develop liver fibrosis, which is the major predictor of subsequent liver cirrhosis, hepatocellular carcinoma and transplantation [4]. Consequently, early detection of fibrosis, before its progression, is important for the prevention and appropriate treatment of these pathologies. To date, liver biopsy has been regarded as the gold standard method of diagnosing and staging liver fibrosis [5, 6]. However, liver biopsy is invasive and is associated with several risks, including high sampling variability, because of the small size of the tissue samples obtained, inter-observer variability, pain and complications due to the procedure itself [7, 8]. A number of non-invasive methods of staging fibrosis and determining the treatment response have been reported in recent years [9–13], but none of these have become well established as diagnostic techniques. The most successful non-invasive approaches in clinical practice are ultrasound- and magnetic resonance-based elastography, which assess liver stiffness [12, 13]. Consequently, they do not provide information regarding the molecular pathology of liver fibrosis [14, 15]. Therefore, the development and validation of a non-invasive biomarker for the diagnosis and staging of liver fibrosis would be valuable.

The pathology of NASH and the mechanism of progression of liver fibrosis have been well documented [16]. The activation of hepatic stellate cells (HSCs) is one of the most important events in liver fibrogenesis [17]. Activated HSCs play an important role in fibrogenesis by secreting proteins such as integrin α_v_β_3_ that play a critical role in the transformation of HSCs to myofibroblasts, which express α-smooth muscle actin (α-SMA). This leads to excessive production of extracellular matrix (ECM) proteins, such as collagens type-1 and type-3 [18]. Therefore, the expression of integrin α_v_β_3_ may have potential as a marker of fibrosis in the liver.

Integrin α_v_β_3_ positron emission tomography (PET) probes, such as ^18^F-labelled RGD [19, 20], have previously been used to detect liver fibrosis in animal models. However, evaluation of the kinetic profile of the binding of ^18^F-FPP-RGD_2_ to integrin α_v_β_3_ in the livers of animal models of NASH is a critical step. There have been previous kinetic analyses of RGD peptide PET probes, such as ^18^F- and ^68^Ga-labelled RGD [21, 22], in animal models of angiogenesis and neoplasia, but not in models of liver fibrosis and NASH. Therefore, we aimed to conduct a kinetic analysis to calculate the parameter *V*_T_ in an animal model of NASH.

In the present study, we have investigated the relationship between the hepatic *V*_T_ of ^18^F-FPP-RGD_2_, calculated using a kinetic analysis, or the standard uptake value between 60 and 90 min (SUV_60–90 min_) and integrin α_v_β_3_ protein expression using PET imaging in a model of diet-induced NASH.

## Methods

### Animals

Male, 7-week-old RccHan®: WIST rats (Wistar Hannover Rcc rats) were obtained from Japan SLC, Inc. (Shizuoka, Japan). They were allowed free access to tap water and fed a normal diet (CE-2; CLEA, Tokyo, Japan) or a choline-deficient, low-methionine high-fat diet (CDHFD; no choline, 45% fat, 0.1% methionine and 1% cholesterol) prepared by Oriental Yeast Co., ltd. (Tokyo, Japan), for 3–4 or 9–10 weeks. The rats were housed at a controlled temperature and under a 12-h light–dark cycle (lights on at 07:00 h). The experiments were approved by the Institutional Animal Care and Use Committee of Osaka University Graduate School of Medicine (approval number: 29-030-002, 21st July 2017).

### Biochemical and histological analysis

After PET/CT scanning, all rats (5 animals/group) were killed by exsanguination under isoflurane anaesthesia. Plasma samples were collected and assayed for aspartate aminotransferase (AST), alanine aminotransferase (ALT) and alkaline phosphatase (ALP) activity, and total cholesterol (TC), triglyceride (TG), glucose (GLU), total bile acid (TBA), albumin (ALB) and total bilirubin (T-BIL) concentrations by enzymatic methods using commercially available kits (Sekisui Medical, Tokyo, Japan) and a Hitachi 7170 autoanalyser (Hitachi, Tokyo, Japan), according to the manufacturer’s instructions.

Liver samples for protein analysis were quickly frozen in liquid nitrogen and stored at − 80 °C until use. The right hepatic lobes were fixed in 10% formalin, routinely processed, and embedded in paraffin. Four-micrometre-thick paraffin sections were prepared, and these were stained with haematoxylin and eosin (H&E) and Sirius red. Steatosis, inflammation and ballooning were graded for severity on H&E-stained sections. Steatosis and inflammation were scored from 0 to 3: normal = 0; minimal = 1; moderate = 2 and marked = 3. Ballooning was scored from 0 to 2: normal = 0; minimal = 1 and marked = 2. NAFLD activity score (NAS) was then calculated as the sum of each of these scores. To assess hepatic fibrosis, Sirius red staining images were captured using a BZ-X700 microscope (Keyence Co., Osaka, Japan) and the Sirius red-positive area (%), corresponding to fibrosis, was measured using the BZ-X analysis application (Keyence Co.).

### Protein analysis

Hepatic integrin α_v_ and β_3_ subunit protein levels were determined using a JESS Automated Western Blotting system (ProteinSimple, San Jose, CA, USA). Liver tissue lysates were prepared in RIPA Lysis and Extraction Buffer (Thermo Fisher Scientific K.K., Tokyo, Japan), and lysates containing 0.2 mg/mL protein were separated using a 12–230 kDa Separation Module (ProteinSimple). Specific proteins were detected using mouse anti-integrin α_v_ (1:50; BD Biosciences, San Jose, CA, USA; ab611012), rabbit anti-integrin β_3_ (1:50; Abcam, Toronto, ON, Canada; ab210515) and an Anti-Mouse Detection Module (ProteinSimple), according to the manufacturer’s instructions.

### PET probe synthesis

^18^F-FPP-RGD_2_ was synthesised using a two-step method, as reported previously [20, 23], to a specific activity of 273.3 ± 60.7 GBq/μmol. The RGD dimeric peptide (PEG_3_-c[RGDyK]_2_) was purchased from Peptides International, Inc. (Louisville, KA, USA).

### Analysis of the metabolites of ^18^F-FPP-RGD_2_ using thin-layer chromatography (TLC)

Metabolite analysis of plasma and liver samples from six normal diet-fed rats was conducted as described previously [24]. Briefly, ~ 20 MBq ^18^F-FPP-RGD_2_ was administered via a tail vein, and then, blood and liver samples were collected 30 and 90 min later under isoflurane anaesthesia. Blood was drawn from the abdominal vena cava; then, the rats were exsanguinated, and their livers were collected and quickly homogenised on ice. Plasma was prepared by 1 min of centrifugation at 4 °C and 20,817×*g*. The plasma and liver samples were deproteinised by precipitation with acetonitrile then centrifuged at 20,817×*g* and 4 °C for 5 min, and the supernatants were applied to RP-18 TLC plates (Merck KGaA, Darmstadt, Germany). The plates were developed at room temperature using 10% ammonium acetate/methanol (50:50) as the mobile phase then dried and used to expose an imaging plate (Fuji Film Corp., Tokyo, Japan) for 30 min. With reference to the Rf value of an ^18^F-FPP-RGD_2_ standard, the distribution of radioactivity for ^18^F-FPP-RGD_2_ on the imaging plates was determined by digital PSL autoradiography using a Typhoon FLA 7000 imaging analyser (GE Healthcare, Uppsala, Sweden), and the data were analysed using Multi-Gauge imaging analysis software (Fuji Film Corp.).

### Dynamic PET imaging

Dynamic PET/CT imaging was performed using a Triumph LabPET-12 PET/CT (TriFoil Imaging Inc., Chatsworth, CA, USA) and a PET camera with an intrinsic axial resolution of 1.38 mm FWHM (full width at half maximum) [25]. Under 2% isoflurane anaesthesia, a tail vein was catheterised for intravenous injection of the PET probe, and the rats were placed on a heated pad on the scanner bed. Normal diet- and CDHFD-fed rats were imaged 3–4 or 9–10 weeks after starting the diets. Five rats per group were used for this experiment. At the start of the PET scan, ^18^F-FPP-RGD_2_ (15.5 ± 2.2 MBq) was administered intravenously at a constant rate of 0.5 mL/30 s via a syringe pump (Legato 210; KD Scientific Inc., Holliston, MA, USA) in all animals. PET scanning was performed in dynamic scan mode for 90 min; then, CT scanning was performed to acquire anatomical information and correct the PET images for attenuation.

For the analysis of the arterial input function (AIF) in the 6 normal diet-fed rats, a femoral artery was also catheterised (insertion length; about 5 cm from the femoral artery) for blood collection under 2% isoflurane anaesthesia, and the cannula was flushed with heparinized saline. Arterial blood sampling was conducted 12 times over the following time periods: 0–10, 10–20, 20–30, 30–40, 40–50, 50–60, 90–100, 300–310, 900–910, 1800–1810, 3600–3610, and 5390–5400 s after ^18^F-FPP-RGD_2_ administration. Once blood sampling was done, the cannula was flushed with heparinized saline for the next sampling. The volume of blood removed during each time period was ~ 50 µL, making the total volume ~ 600 µL, which was ~ 5% of the total blood volume. Plasma was prepared by 1 min of centrifugation at 4 °C and 20,817×*g*. The radioactivity of the plasma was measured using a gamma counter (2480 Wizard^2^, PerkinElmer, Inc., Waltham, MA, USA) and was expressed as counts per minute/mL (cpm/mL). Each radioactive count was corrected for decay since the start of the gamma counting and was converted into an SUV.

### Image processing and kinetic analysis

CT images were reconstructed using the filtered back-projection method (512 slices), and PET images were reconstructed into 25 frames of increasing length (6 × 10 s, 4 × 60 s, 11 × 300 s and 3 × 600 s) using the three-dimensional maximum-likelihood expectation maximisation (3D-MLEM) algorithm and CT-based attenuation correction. To obtain time-activity curves (TACs) for kinetic analysis, the left ventricle (33 mm^3^) and liver (1280 mm^3^) volumes of interest (VOIs) were manually defined for each animal on their CT images using PMOD PET data analysis software (v3.905, PMOD Technologies Ltd., Zurich, Switzerland). The VOI size was same in all animals. A spherical VOI on the left ventricle was used to obtain an image-derived plasma input function. TACs for the left ventricle and the liver were constructed by normalising decay-corrected time activity measurements to the injected doses of ^18^F-FPP-RGD_2_ and are expressed as mean standardised uptake values (SUVs), where SUV = radioactivity concentration (kBq/cm^3^) × body mass (g)/amount of radioactivity injected (MBq)/1000.

**Fig. 1:**
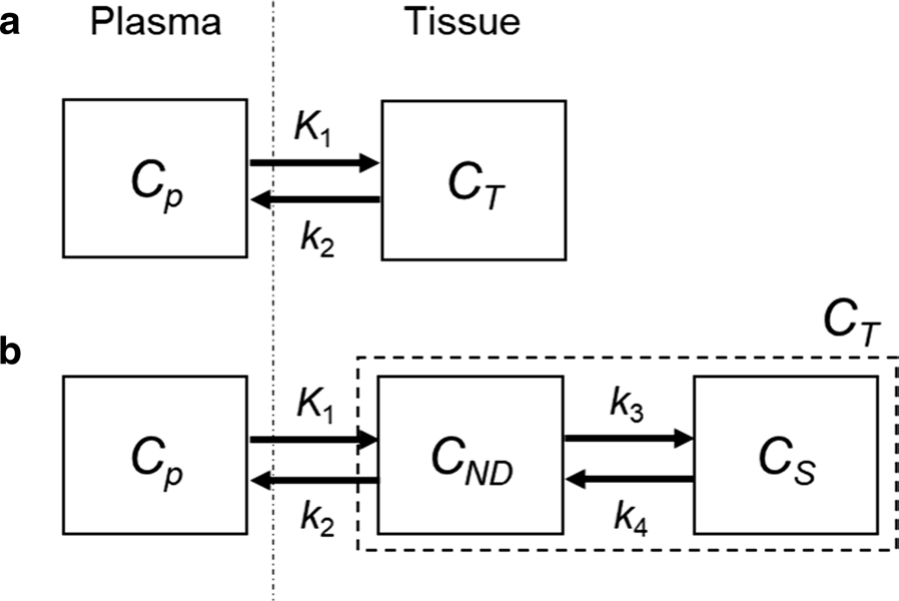
Compartment models for kinetic analysis. **a** a one-tissue, two-compartment (1T2C) and **b** a two-tissue, three-compartment (2T3C) model

Kinetic modelling to determine the total distribution volume (*V*_T_) of ^18^F-FPP-RGD_2_ was performed by fitting the TAC using a one-tissue, two-compartment (1T2C) and a two-tissue, three-compartment (2T3C) model using the PMOD software [26]. Figure 1 shows that (a) 1T2C and (b) 2T3C model in which, *C*_p_, *C*_T_, *C*_ND_ and* C*_S_ represent the PET probe concentration of the plasma, total binding to integrin α_v_β_3_, free or non-specific binding and specific binding to integrin α_v_β_3_, respectively. *K*_1_, *k*_2_, *k*_3_ and *k*_4_ represent the extravasation rate of the PET probe, tissue efflux rate of free or non-specific binding probe, the rate of specific binding of PET probe to the extracellular portion of the integrin α_v_β_3_ and dissociation rate, respectively. A physiological parameter of interest (*V*_T_; volume of distribution) was calculated for the tissue of interest. *V*_T_ is a measure of the total (i.e. both non-displaceable and specific binding) distribution of the radioligand into the tissue and is equivalent to an equilibrium partition coefficient. *V*_*T*_ was calculated by the following equation:

$$V_{{\text{T}}} = V_{{{\text{ND}}}} + V_{{\text{S}}} = \frac{{K_{1} }}{{k_{2} }}\left( {1 + \frac{{k_{3} }}{{k_{4} }}} \right)$$

The Akaike information criterion (AIC) and curve fitting were used to determine the most appropriate compartment model for ^18^F-FPP-RGD_2_. AIC calculated by PMOD software was commonly used as statistic criteria [27] to compare the data fitting between different models [22, 28, 29], where lowest AIC value provides the most appropriate model. When we compared the *V*_T_ calculated using the AIF and the *V*_T_ calculated using the image-derived input function (IDIF), there is a stronger linear correlation between *V*_T (AIF)_ and *V*_T (IDIF)_ (Spearman's rank correlation *r*_s_ = 0.943, *P* < 0.05) in the present study; therefore, IDIF was used in the present study. Models with irreversible binding were not considered because of the reversibility of the binding of this probe indicated by tissue TACs.

### Statistical analysis

Comparisons of the two and four groups of rats were made using unpaired Student’s *t* tests with Welch’s correction and Steel–Dwass test, respectively. Spearman's rank correlation was used to evaluate the relationships between two variables (integrin α_v_ or β_3_ protein expression and SUV_60–90 min_, *V*_T (IDIF)_, *V*_T (IDIF)_ and *V*_T (AIF)_). Statistical analyses were performed using GraphPad Prism (version 6) statistical software (GraphPad Software, San Diego, CA), and SAS studio (version 3.71). *P* < 0.05 was considered to indicate statistical significance (two-tailed).

## Results

### Biochemical and histological evaluation

Body and liver mass, liver/body mass ratio and biochemical parameters are shown in Table 1. The body masses of the CDHFD-fed rats were significantly lower than those of the normal diet-fed rats after 3–4 and 9–10 weeks, whereas the liver masses and liver/body mass ratios were significantly higher. The AST and TBA of the 9–10-week CDHFD-fed group were significantly higher than those of the normal diet-fed group, and the ALT, ALP and T-BIL of both the 3–4- and 9–10-week CDHFD-fed groups were significantly higher than those of the normal diet-fed groups. The ALB of the 9–10-week CDHFD-fed group was significantly lower than that of the normal diet-fed group, and the TG and GLU of both the 3–4- and 9–10-week CDHFD-fed groups were significantly lower than those of the normal diet-fed groups.

**Table 1:** Body and liver masses, liver/body mass ratio and biochemical parameters

Parameters	Group			
	3–4 weeks		9–10 weeks	
	Normal diet	CDHFD	Normal diet	CDHFD
Body weight (g)	299.1 ± 20.5	206.2 ± 15.7**	433.3 ± 16.2	246.0 ± 11.9**
Liver weight (g)	9.9 ± 0.8	13.9 ± 2.1**	12.1 ± 12	18.8 ± 2.9**
Liver/body weight ratio	0.033 ± 0.001	0.067 ± 0.007**	0.028 ± 0.002	0.076 ± 0.008**
AST (IU/L)	74.9 ± 12.6	564 ± 438	62.7 ± 16.6	566 ± 229**
ALT (IU/L)	45.1 ± 13.1	760 ± 528*	38.6 ± 15.9	229 ± 94.6*
TC (mg/dL)	56.4 ± 4.4	31.4 ± 3.1	51.7 ± 10.4	66.6 ± 23.7
TG (mg/dL)	96.2 ± 33.4	4.6 ± 3.2**	184 ± 131	8.1 ± 4.6*
GLU (mg/dL)	192 ± 20.1	143 ± 13.8**	201 ± 18.1	127 ± 16.6**
TBA (mg/dL)	7.6 ± 4.6	117 ± 91.3	13.2 ± 12.2	138 ± 75.1*
ALB (g/dL)	2.2 ± 0.1	2.3 ± 0.1	2.3 ± 0.1	2.1 ± 0.2*
ALP (IU/L)	518 ± 75.7	1109 ± 427*	316 ± 69.7	1757 ± 818*
T-BIL (mg/dL)	0.11 ± 0.02	0.44 ± 0.24*	0.09 ± 0.02	0.66 ± 0.41*

Values are expressed as means ± SDs (*n* = 5). *P < 0.05, **P < 0.01, compared with the respective normal diet-fed group

*CDHFD* choline-deficient, low-methionine high-fat diet, *AST* aspartate aminotransferase, *ALT* alanine aminotransferase, *TC* total cholesterol, *TG* triglyceride, *GLU* glucose, *TBA* total bile acids, *ALB* albumin, *ALP* alkaline phosphatase, *T-BIL* total bilirubin

**Fig. 2:**
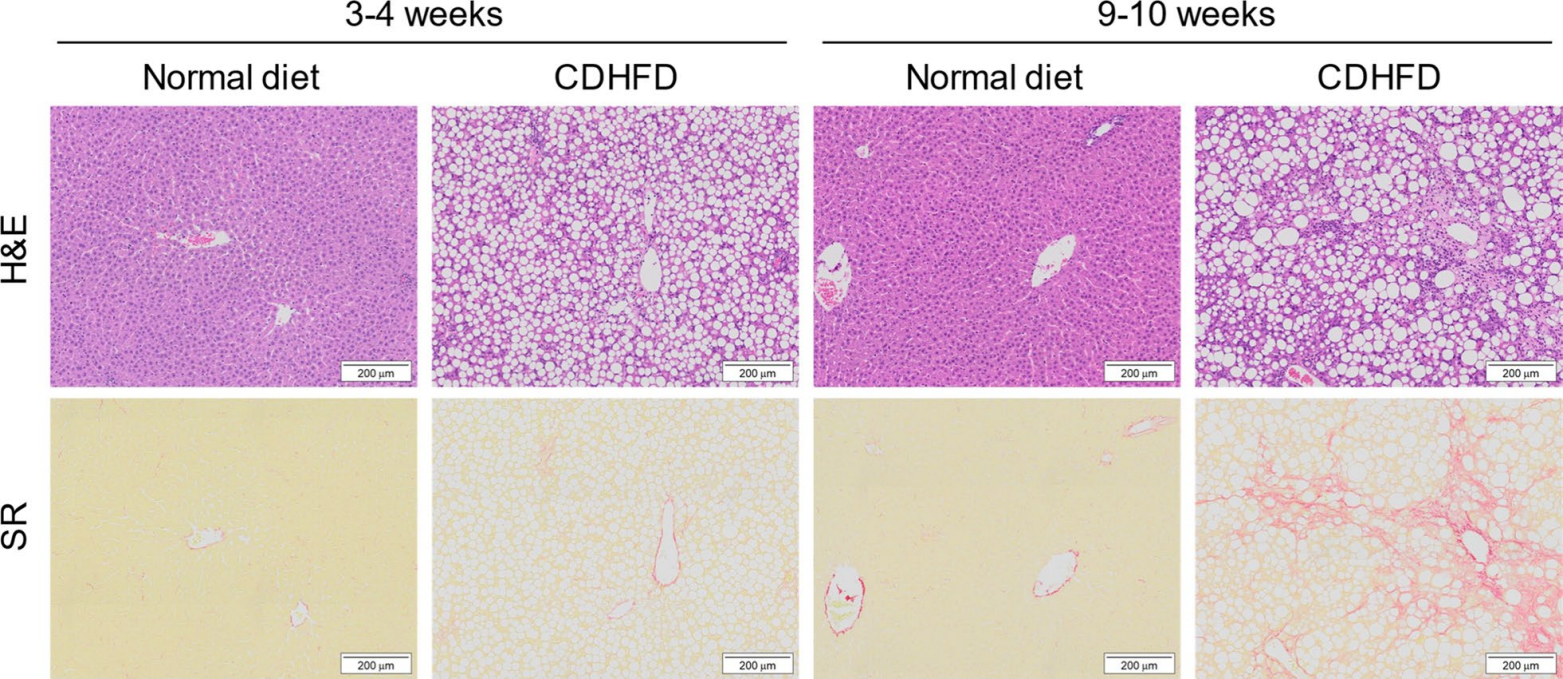
Representative photomicrographs of hepatic histopathology in H&E- and Sirius red-stained sections. *CDHFD* choline-deficient, low-methionine high-fat diet

Figure 2 presents the representative photomicrographs of hepatic histopathology in H&E- and Sirius red-stained sections. As shown in Table 2, histopathological analysis demonstrated that steatosis and inflammation were induced by 3–4 and 9–10 weeks of CDHFD consumption (steatosis score: 3–4 weeks, *P* < 0.01 and 9–10 weeks, *P* < 0.01; inflammation score: 3–4 weeks, *P* < 0.01 and 9–10 weeks,* P* < 0.01), and ballooning was induced by 9–10 weeks of CDHFD consumption (*P* < 0.01). In addition, the inflammation (*P* < 0.01) and ballooning (*P* < 0.05) scores were higher in 9–10-week CDHFD-fed rats than in 3–4-week CDHFD-fed rats. The NAFLD activity score and histopathological NASH diagnostic criteria in both 3–4- and 9–10-week CDHFD-fed groups were significantly higher than those in the respective normal diet-fed groups (*P* < 0.01), and the scores in the 9–10-week CDHFD-fed group were higher than those in the 3–4-week CDHFD-fed group (*P* < 0.01). The area of fibrosis in the 9–10-week CDHFD-fed group was significantly higher than those in the normal diet-fed (*P* < 0.05) and 3–4-week CDHFD-fed (*P* < 0.05) groups, whereas the area of fibrosis in the 3–4-week CDHFD-fed group was not significantly larger than that in the 3–4-week normal diet-fed group.

**Table 2:** Histological parameters, evaluated using H&E and SR staining

Parameters	Group			
	3–4 weeks		9–10 weeks	
	Normal diet	CDHFD	Normal diet	CDHFD
Steatosis score	0.0 ± 0.0	3.0 ± 0.0 **	0.0 ± 0.0	3.0 ± 0.0 **
Inflammation score	0.0 ± 0.0	1.4 ± 0.55 **	0.20 ± 0.45	2.6 ± 0.55 **^, ††^
Ballooning score	0.0 ± 0.0	0.20 ± 0.45	0.0 ± 0.0	1.0 ± 0.0 **^, †^
NAFLD activity score	0.0 ± 0.0	4.6 ± 0.89 **	0.20 ± 0.45	6.6 ± 0.55 **^, ††^
Fibrosis area (%)	1.0 ± 0.0	1.4 ± 0.5	1.0 ± 0.0	19.8 ± 10 *^, †^

Values are expressed as means ± SDs (*n* = 5). *P < 0.05, **P < 0.01, compared with the respective normal diet-fed groups; ^†^*P* < 0.05; ^††^*P* < 0.01, compared with the 3–4-week CDHFD-fed group

*H&E* haematoxylin and eosin, *SR* Sirius red, *CDHFD* choline-deficient, low-methionine high-fat diet, *NAFLD* non-alcoholic fatty liver disease

### Hepatic integrin α_v_ and β_3_ expression

**Fig. 3:**
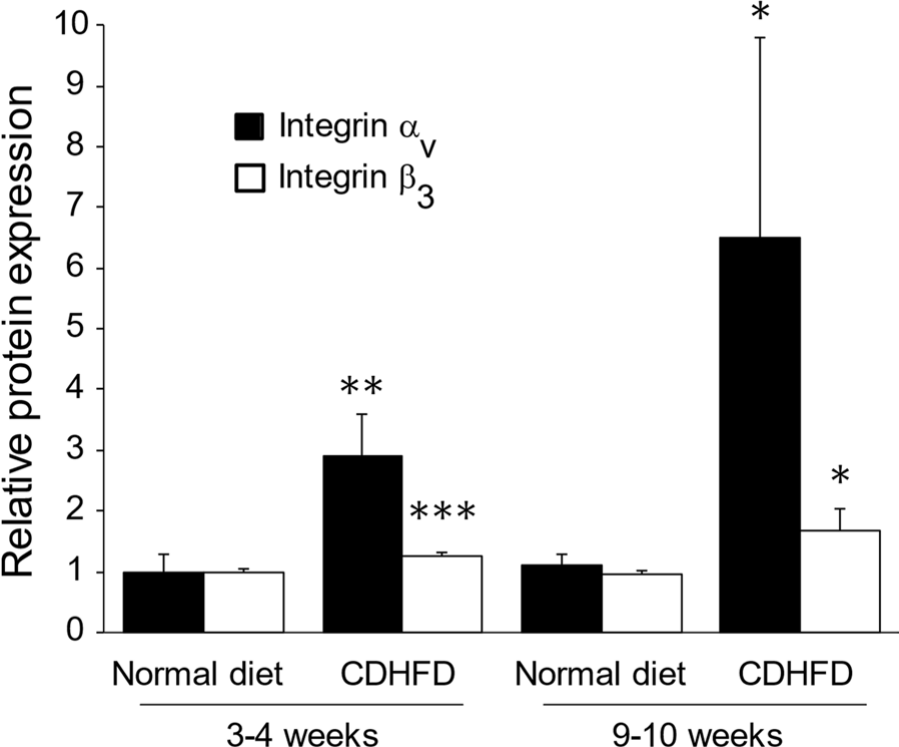
Hepatic integrin α_v_ and β_3_ protein expression. Values are protein expression relative to that of the mean value for the 3–4-week normal-diet-fed group; means ± SDs (*n* = 5). The vertical axis values are the integrin α_v_ or β_3_ protein expression relative to that of the mean of the 3–4-week normal diet-fed group. *P < 0.05; **P < 0.01; ***P < 0.001 compared with the equivalent normal diet-fed groups. *CDHFD* choline-deficient, low-methionine high-fat diet

As shown in Fig. 3, the integrin α_v_ and β_3_ protein expression in the CDHFD-fed groups was significantly higher than that in the normal diet-fed groups (3–4 weeks: α_v_, *P* < 0.01; β_3_, *P* < 0.001; 9–10 weeks: α_v_, *P* < 0.05; β_3_, *P* < 0.05). Moreover, the integrin α_v_ and β_3_ protein expression in the 9–10-week CDHFD-fed group tended to be higher than that in the 3–4-week CDHFD-fed group.

### Metabolic stability of ^18^F-FPP-RGD_2_

The metabolic stability of ^18^F-FPP-RGD_2_ in the plasma and liver was evaluated by TLC autoradiography in normal diet-fed animals. Additional file 1: Table 1 shows the mean non-metabolised percentages of ^18^F-FPP-RGD_2_ in the plasma and liver 30 and 90 min after ^18^F-FPP-RGD2 was intravenously administered. The mean non-metabolised percentage in all of the rats was > 96%, and there were no significant radioactive signals on the imaging plates corresponding to metabolites (Additional file 1: Fig. 1), suggesting that ^18^F-FPP-RGD_2_ is not readily metabolised.

### Hepatic uptake of ^18^F-FPP-RGD_2_

**Fig. 4:**
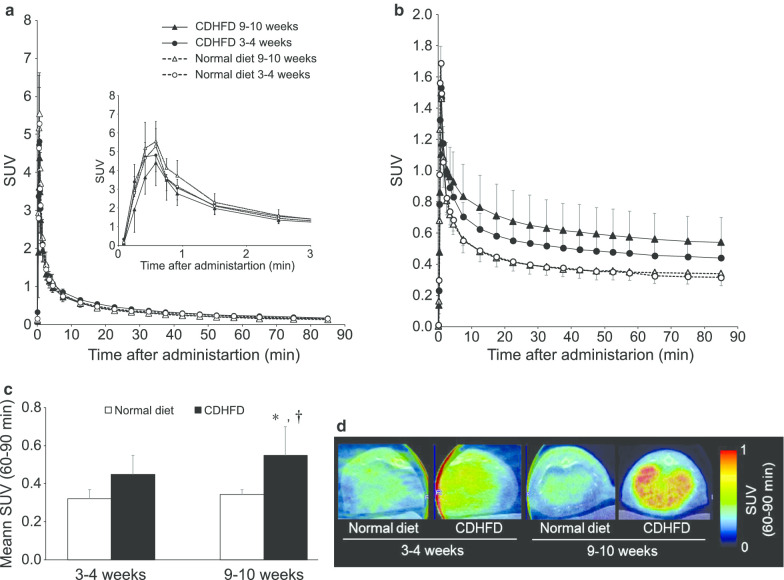
SUV profile and representative colour-coded PET images. **a** Left ventricular time-activity curve and **b** hepatic time-activity curve after ^18^F-FPP-RGD_2_ administration. **c** Mean SUV and **d** representative PET/CT fusion images 60–90 min after ^18^F-FPP-RGD_2_ administration. Values are expressed as means ± SDs (*n* = 5). *P < 0.05 compared with the 3–4-week normal diet-fed group; ^†^*P* < 0.05 compared with 3–4-week CDHFD-fed group. *CDHFD* choline-deficient, low-methionine high-fat diet

Left ventricular SUV peaked within 1 min of the administration of ^18^F-FPP-RGD_2_, then was rapidly eliminated, such that approximately constant values were reached at 60–90 min in all the groups (Fig. 4a). Therefore, the mean hepatic SUV was calculated using the SUVs between 60 and 90 min after administration. Figure 4b, c shows the hepatic TACs and SUVs, respectively, 60–90 min (SUV_60–90 min_) after the administration of ^18^F-FPP-RGD_2_. The SUV_60–90 min_ of the 3–4- and 9–10-week CDHFD-fed groups were higher than those of the normal diet-fed groups (Fig. 4c). In addition, the SUV_60–90 min_ of the 9–10-week CDHFD-fed group was significantly higher than that of the 3–4-week normal diet-fed and CDHFD-fed group (*P* < 0.05, Fig. 4c). The SUV_60–90 min_ of the 3–4- and 9–10-week normal diet-fed groups were almost identical (Fig. 4c). Figure 3d shows representative colour-coded PET images of the livers of rats that had consumed normal diets or CDHFD for 3–4 or 9–10 weeks.

### Kinetic analysis

**Fig. 5:**
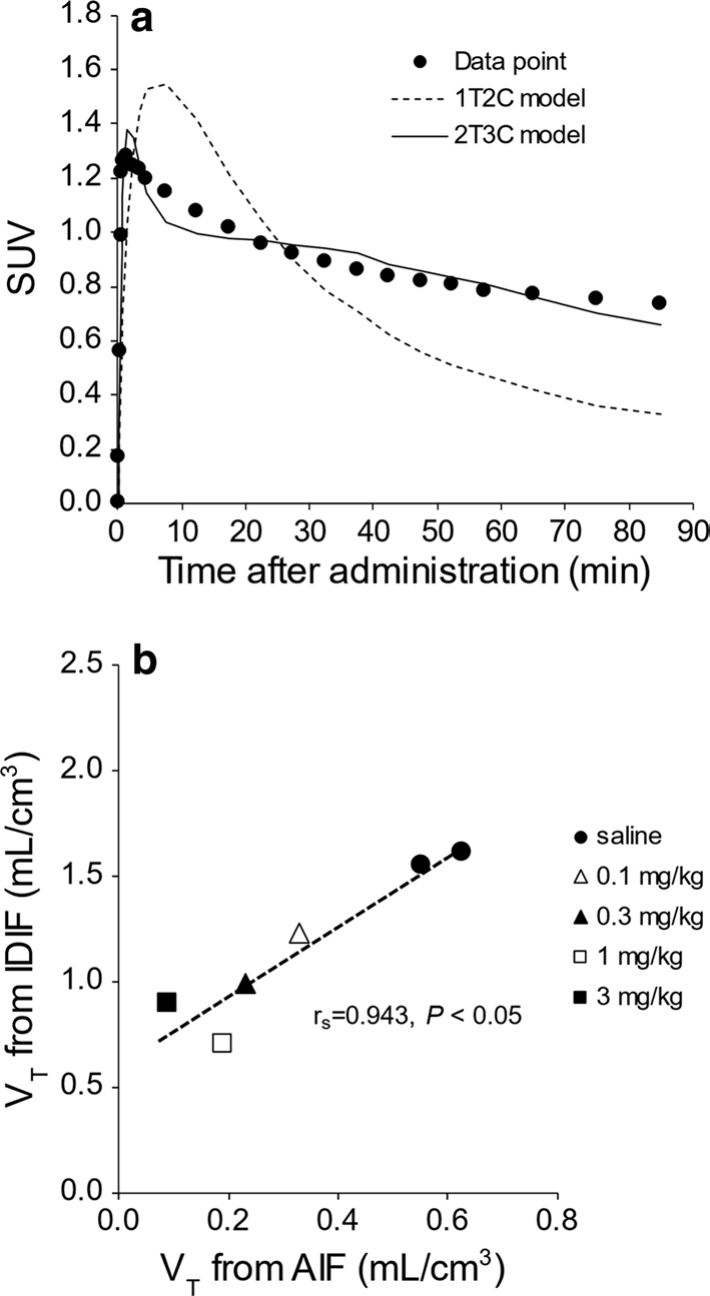
Curve fitting for the compartment analysis and correlation analysis with *V*_T_. **a** Quality of curve fitting for a representative hepatic time-activity curve and **b** correlation between *V*_T_ obtained using IDIF and *V*_T_ obtained using AIF. Saline or unlabelled RGD (0.1, 0.3, 1, or 3 mg/kg) were administered intravenously 5 min before ^18^F-FPP-RGD_2_. *1T2C model* one-tissue two-compartment model, *2T3C model* two-tissue three-compartment model, *IDIF* image-derived input function, *AIF* arterial input function

**Fig. 6:**
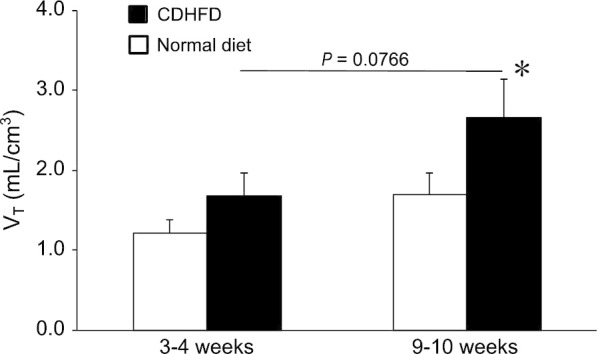
Hepatic *V*_T_ analysed by 2T3C model analysis. Values are expressed as means ± SDs (*n* = 5). *P < 0.05 compared with the 3–4-week normal diet-fed group. *CDHFD* choline-deficient, low-methionine high-fat diet

Table 3 shows the rate constants (*K*_1_, *k*_2_, *k*_3_, and *k*_4_) and AICs obtained from the kinetic analysis using the 1T2C and 2T3C models in normal diet- and CDHFD-fed rats after 3–4 and 9–10 weeks. The AIC associated with the 2T3C model was lower than that associated with the 1T2C model for all the groups. As shown in Fig. 5a, the quality of the curve fitting also indicated that the 2T3C model was more appropriate than the 1T2C model for the kinetic analysis of ^18^F-FPP-RGD_2_ in the liver. To confirm the accuracy of IDIF, we evaluated the relationships between *V*_T_ obtained using IDIF and the *V*_T_ obtained using AIF in normal diet-fed rats, with or without inhibition using unlabelled RGD (0.1–3 mg/kg). The *V*_T (IDIF)_ strongly correlated with the *V*_T (AIF)_ (Spearman’s rank correlation, *r*_s_ = 0.943, *P* < 0.05, Fig. 5b). Figure 6 shows the *V*_T (IDIF)_ for ^18^F-FPP-RGD_2_ calculated using the 2T3C model. The *V*_T (IDIF)_ was higher in both the 3–4- and 9–10-week CDHFD-fed groups than in the normal diet-fed groups and increased with the duration of CDHFD feeding (Fig. 6). In addition, the *V*_T (IDIF)_ of the 9–10-week CDHFD-fed group was significantly higher than that of the 3–4-week normal diet-fed group (*P* < 0.05, Fig. 6).

**Table 3:** Kinetic parameters, determined using compartment analysis

Group	Model	*K*_1_	*k*_2_	*k*_3_	*k*_4_	AIC
*3–4 weeks*						
Normal diet	1T2CM	1.06 ± 0.26	2.08 ± 0.40	–	–	89.33 ± 4.8
	2T3CM	1.43 ± 0.34	3.63 ± 0.87	0.05 ± 0.012	0.03 ± 0.0063	35.96 ± 3.8
CDHFD	1T2CM	0.74 ± 0.14	1.15 ± 0.32	–	–	93.31 ± 10.7
	2T3CM	0.90 ± 0.12	1.94 ± 0.47	0.05 ± 0.020	0.02 ± 0.0021	41.14 ± 3.9
*9–10 weeks*						
Normal diet	1T2CM	0.87 ± 0.34	1.56 ± 0.85	–	–	97.59 ± 3.8
	2T3CM	1.26 ± 0.14	3.00 ± 0.40	0.05 ± 0.0075	0.02 ± 0.0048	43.93 ± 5.9
CDHFD	1T2CM	0.42 ± 0.26	0.51 ± 0.61	–	–	100.3 ± 7.9
	2T3CM	0.69 ± 0.14	1.19 ± 0.39	0.07 ± 0.0079	0.02 ± 0.0029	32.12 ± 7.8

Values are expressed as means ± SDs (*n* = 5)

*CDHFD* choline-deficient, low-methionine high-fat diet, *1T2CM* one-tissue two-compartment model, *2T3CM* two-tissue three-compartment model, *AIC* Akaike information criterion

### Relationships between SUV_60–90 min_ or *V*_T_ and integrin protein expression

**Fig. 7:**
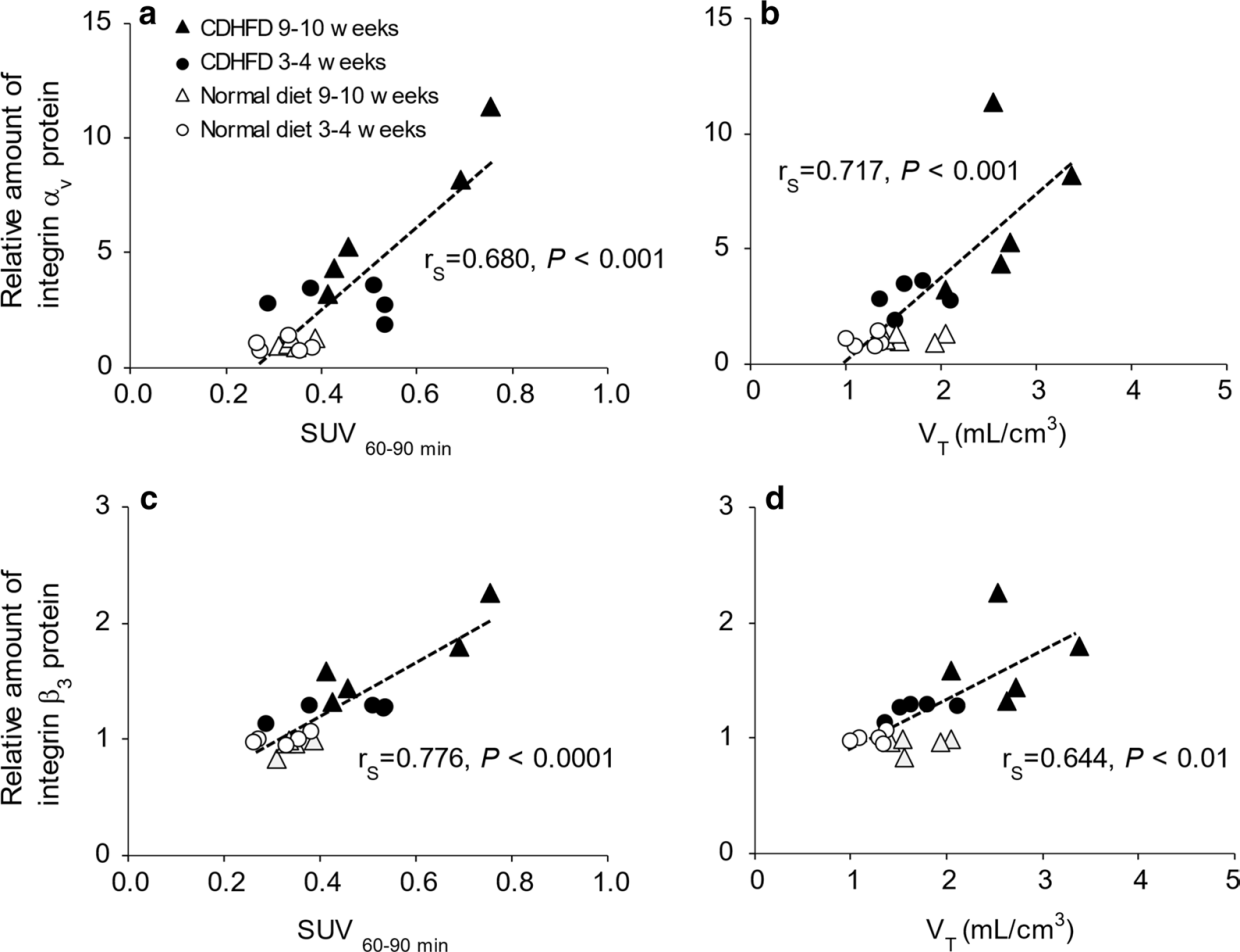
Correlation analysis of SUV_60–90 min_ or *V*_T_ and integrin. Correlation between SUV_60–90 min_ and **a** integrin α_v_ and **c** β_3_ protein expression. Correlation between *V*_T_ obtained using the 2T3C model and **b** integrin α_v_ and **d** β_3_ protein expression. Vertical axis values are the integrin α_v_ or β_3_ protein expression relative to the mean value for the 3–4-week normal diet-fed group. *CDHFD* choline-deficient, low-methionine high-fat diet

Figure 7 shows the correlations between SUV_60–90 min_ or *V*_T (IDIF)_ and integrin α_v_ or β_3_ protein expression. Both SUV_60–90 min_ and *V*_T (IDIF)_ strongly correlated with integrin α_v_ and β_3_ protein expression (Spearman's rank correlation, SUV_60–90 min_ vs. α_v_, *r*_s_ = 0.680, *P* < 0.001; SUV_60–90 min_ vs. β_3_, *r*_s_ = 0.776, *P* < 0.0001; *V*_T_ vs. α_v_, *r*_s_ = 0.717, *P* < 0.001; *V*_T_ vs. β_3_, *r*_s_ = 0.644, *P* < 0.01).

## Discussion

In the present study, we have evaluated the kinetics of ^18^F-FPP-RGD_2_ in an animal model of NASH, as a means of quantifying hepatic integrin α_v_β_3_ protein expression. We found strong correlations between hepatic *V*_T (IDIF)_, evaluated using the 2T3C model or SUV_60–90 min_, and hepatic integrin α_v_ or β_3_ protein expression. The present findings indicate that higher hepatic *V*_T (IDIF)_ values for ^18^F-FPP-RGD_2_ may be a predictor of fibrosis associated with NASH.

The CDHFD-fed rat is a suitable model of NASH because it demonstrates clinically relevant onset and progression of hepatic fibrosis [30, 31]. In the present study, histological and biochemical analysis confirmed the progression of NASH pathology, and the severity of the pathological changes increased alongside increases in hepatic integrin α_v_ and β_3_ protein expression. These findings were in accordance with those of previous studies conducted in similar animal models [20, 30, 32]. Histopathological examination confirmed the development of NASH and fibrosis in CDHFD-fed rats. As shown by Kleiner et al. [33], CDHFD-feeding for 3–4 or 9–10 weeks caused borderline NASH and frank NASH, according to the NAFLD activity score. In addition, the area of fibrosis in the 9–10-week CDHFD-fed group was significantly larger than that in the normal diet-fed group, whereas the area of fibrosis in the 3–4-week CDHFD-fed group was not significantly higher than that in the 3–4-week normal diet-fed group. This indicates that 3–4 weeks of CDHFD feeding results in the development of NAFLD/NASH with minimal or no fibrosis and that 9–10 weeks of feeding results in NASH with moderate fibrosis.

HSC activation plays a pivotal role not only in the onset, but also in the progression of hepatic fibrosis [17]. The integrin α_v_β_3_ expression level is low in quiescent HSCs, hepatocytes and non-parenchymal cells [34]. On the other hand, many several studies of liver fibrosis revealed that integrin α_v_β_3_ expression level is upregulated on activated HSC [35–38]. Therefore, integrin α_v_β_3_ has been shown to be a biomarker of HSC activation [39]. In the present study, hepatic integrin α_v_ and β_3_ protein expression in CDHFD-fed rats was higher from 3–4 weeks of feeding, prior to the development of significant fibrosis. Therefore, it seems that integrin α_v_β_3_ protein expression increases, indicating HSC activation, before the development of fibrosis in rats with CDHFD-induced NASH.

The metabolite analysis confirmed that there is negligible metabolism of ^18^F-FPP-RGD_2_ in the plasma and liver up to 90 min after intravenously administration. Hepatic TAC fitting revealed that the 2T3C model was more appropriate than the 1T2C model, and this was also quantitatively confirmed using the AIC values. Consistent with the results of a previous study [3], hepatic TACs for ^18^F-FPP-RGD_2_ peaked rapidly after administration and then gradually decreased until the final time point, implying that ^18^F-FPP-RGD_2_ kinetics are consistent with a reversible receptor-binding model. Therefore, in the present study, we did not evaluate an irreversible compartment model. These data indicate that ^18^F-FPP-RGD_2_ binds to integrin α_v_β_3_ proteins reversibly and that a 2T3C model is suitable for the analysis of ^18^F-FPP-RGD_2_ kinetics. Moreover, we found that there is a strong correlation (Spearman's rank correlation *r*_s_ = 0.943, *P* < 0.05) between the *V*_T (IDIF)_ and *V*_T (AIF)_. Therefore, the ^18^F-FPP-RGD_2_ activity in left ventricular blood as IDIF was used for kinetic analysis, instead of AIF, with the assumption that there was no inter-individual variation in haematocrit.

^18^F-labelled RGD, which binds to integrin α_v_β_3_, has previously been used to detect liver fibrosis in animal models [19, 20]. A previous study showed the colocalisation of integrin α_v_β_3_ with α-SMA in activated HSCs by immunofluorescence staining and that fluorescently labelled RGD binds to activated HSCs [34]. In the present study, the hepatic SUV or *V*_T (IDIF)_ for ^18^F-FPP-RGD_2_ positively correlated with hepatic integrin α_v_ and β_3_ protein expression and the hepatic *V*_T_ of ^18^F-FPP-RGD_2_ was reduced by the co-administration of unlabelled RGD. These findings indicate that ^18^F-FPP-RGD_2_ specifically binds to hepatic integrin α_v_β_3_ on HSCs in a rat model of NASH.

Next, we conducted 90-min dynamic PET scans using ^18^F-FPP-RGD_2_ and determined the most suitable kinetic model for the evaluation of the liver in rats with CDHFD-induced NASH. In the 3–4-week CDHFD-fed groups, the image-derived input function was similar to that of the normal diet-fed groups. However, in the 9–10-week CDHFD-fed group, although there was no difference in the image-derived input function during the late phase, it was slightly lower during the early phase (0–1 min after administration). Moreover, the *K*_1_, extravasation rate of the PET probe in the CDHFD-fed groups was lower than in the respective normal diet-fed groups. The hepatic SUVs of the CDHFD groups were higher than those of the respective normal diet-fed groups and significantly increased with the duration of the feeding period. These data indicate that hepatic integrin α_v_β_3_ protein expression in CDHFD-fed rats is higher than that in normal diet-fed rats. The *V*_T (IDIF)_ for ^18^F-FPP-RGD_2_, evaluated using the 2T3C model, was higher in the CDHFD-fed groups than in the normal diet-fed groups and strongly positively correlated with hepatic integrin α_v_ (*r*_s_ = 0.717) and β_3_ (*r*_s_ = 0.644) protein expression. The strengths of these correlations were similar to those between SUV_60–90 min_ and hepatic integrin α_v_ (*r*_s_ = 0.680) or β_3_ (*r*_s_ = 0.776) protein expression. Indeed, the clinical usefulness of *V*_T_ has recently been reported for pulmonary fibrosis related to other causes than cancer. Both idiopathic pulmonary fibrosis/healthy control *V*_T_ ratio and SUV ratio > 1. Thus, the *V*_T_ and SUV of [^18^F] FB-A20FMDV2 were quantify expression of integrin α_v_β_6_ in the lungs of subjects with fibrotic interstitial lung disease.[40]. Our data suggest that hepatic *V*_T_, obtained non-invasively using kinetic modelling, might be useful as a predictor of fibrosis and for determining the efficacy of new drugs in pre-clinical and clinical studies. Moreover, the correlation analyses (Fig. 7) suggest that hepatic SUV, which can be easily obtained using a static scan in the clinic, could be used as a predictor of fibrosis with similar efficacy to hepatic *V*_T_ obtained using a dynamic scan.

This study has two limitations. The first is delay or dispersion of input function. Several studies revealed that portal hypertension was induced in NASH patients and diet-induced animal models [41, 42], which lead to cardiovascular dysfunction, such as arterial blood pressure and blood volume decrease [43]. Moreover, the cardiac failure was developed in patients of cirrhosis, due to continuous of hyperdynamic state [44–46]. Our NASH model also might develop cardiovascular dysfunction and have the decreased input function. But we have estimated the fitting without these points. The hepatic blood supply is derived from two vessels: the hepatic artery and the portal vein. However, in the present study, the left ventricular time-activity curves were used as the IDIF without considering the input function from the portal vein. Although the hepatic *V*_T (IDIF)_ of ^18^F-FPP-RGD_2_ strongly positively correlated with hepatic integrin α_v_ and β_3_ protein expression in the present study, further studies are required to evaluate the *V*_T (IDIF)_ using input functions from both the hepatic artery and portal vein.

## Conclusions

Kinetic modelling studies using dynamic ^18^F-FPP-RGD_2_ PET scans are feasible, on the basis of an image-derived input function, in a diet-induced animal model of NASH. Moreover, the kinetic modelling analysis performed in this study will be useful for the quantitative evaluation of ^18^F-FPP-RGD_2_ binding to hepatic integrin α_v_β_3_ proteins. The present findings indicate that higher hepatic *V*_T_ values for ^18^F-FPP-RGD_2_ may offer novel strategies for the prediction of fibrosis associated with NASH.

## Supplementary information

**Additional file 1. Fig. 1:** TLC autoradiograms of plasma and liver extracts from rats 30 or 90 min after the intravenous administration of ^18^F-FPP-RGD2; **Table 1:** Percentages of non-metabolised ^18^F-FPP-RGD2 in the plasma and liver.

## Data Availability

All data generated or analysed during this study are included in this published article and its supplementary information files.

## References

[1] Argo CK, Caldwell SH (2009). Epidemiology and natural history of non-alcoholic steatohepatitis. Clin Liver Dis.

[2] Angulo P (2007). GI epidemiology: nonalcoholic fatty liver disease. Aliment Pharmacol Ther.

[3] Muthiah MD, Sanyal AJ (2020). Burden of disease due to nonalcoholic fatty liver disease. Gastroenterol Clin N Am.

[4] El Serafy MA, Kassem AM, Omar H, Mahfouz MS, El Said EL, Raziky M (2017). APRI test and hyaluronic acid as non-invasive diagnostic tools for post HCV liver fibrosis: systematic review and meta-analysis. Arab J Gastroenterol.

[5] Angulo P (2002). Nonalcoholic fatty liver disease. N Engl J Med.

[6] Talwalkar JA (2002). Motion—all patients with NASH need to have a liver biopsy: arguments for the motion. Can J Gastroenterol.

[7] Cadranel JF, Rufat P, Degos F (2000). Practices of liver biopsy in France: results of a prospective nationwide survey. Hepatology.

[8] Bedossa P, Dargère D, Paradis V (2003). Sampling variability of liver fibrosis in chronic hepatitis C. Hepatology.

[9] Obmann VC, Mertineit N, Berzigotti A, Marx C, Ebner L, Kreis R (2018). CT predicts liver fibrosis: prospective evaluation of morphology- and attenuation-based quantitative scores in routine portal venous abdominal scans. PLoS ONE.

[10] Zhou Y, Chen H, Ambalavanan N, Liu G, Antony VB, Ding Q (2015). Noninvasive imaging of experimental lung fibrosis. Am J Respir Cell MolBiol.

[11] Montesi SB, Désogère P, Fuchs BC, Caravan P (2019). Molecular imaging of fibrosis: recent advances and future directions. J Clin Invest.

[12] Singh S, Venkatesh SK, Wang Z, Miller FH, Motosugi U, Low RN (2015). Diagnostic performance of magnetic resonance elastography in staging liver fibrosis: a systematic review and meta-analysis of individual participant data. Clin Gastroenterol Hepatol.

[13] Herrmann E, de Lédinghen V, Cassinotto C, Chu WCW, Leung VYF, Ferraioli G (2018). Assessment of biopsy-proven liver fibrosis by two-dimensional shear wave elastography: an individual patient data-based meta-analysis. Hepatology.

[14] Petitclerc L, Sebastiani G, Gilbert G, Cloutier G, Tang A (2017). Liver fibrosis: review of current imaging and MRI quantification techniques. J Magn Reson Imaging.

[15] Baues M, Dasgupta A, Ehling J, Prakash J, Boor P, Tacke F (2017). Fibrosis imaging: current concepts and future directions. Adv Drug Deliv Rev.

[16] Schuppan D, Surabattula R, Wang XY (2018). Determinants of fibrosis progression and regression in NASH. J Hepatol.

[17] Tsuchida T, Friedman SL (2017). Mechanisms of hepatic stellate cell activation. Nat Rev Gastroenterol Hepatol.

[18] Friedman SL (2003). Liver fibrosis—from bench to bedside. J Hepatol.

[19] Hartimath SV, Boominathan R, Soh V, Cheng P, Deng X, Chong YC (2019). Imaging fibrogenesis in a diet-induced model of nonalcoholicsteatohepatitis (NASH). Contrast Media Mol Imaging.

[20] Rokugawa T, Konishi H, Ito M, Iimori H, Nagai R, Shimosegawa E (2018). Evaluation of hepatic integrin αvβ3 expression in non-alcoholic steatohepatitis (NASH) model mouse by 18F-FPP-RGD2 PET. EJNMMI Res.

[21] Guo N, Lang L, Li W, Kiesewetter DO, Gao H, Niu G (2012). Quantitative analysis and comparison study of [18F]AlF-NOTA-PRGD2, [18F]FPPRGD2 and [68Ga]Ga-NOTA-PRGD2 using a reference tissue model. PLoS ONE.

[22] Kim JH, Kim Y-H, Kim YJ, Yang BY, Jeong JM, Youn H (2013). Quantitative positron emission tomography imaging of angiogenesis in rats with forelimb ischemia using (68)Ga-NOTA-c(RGDyK). Angiogenesis.

[23] Haskali MB, Roselt PD, Karas JA, Noonan W, Wichmann CW, Katsifis A (2013). One-step radiosynthesis of 4-nitrophenyl 2-[(18) F]fluoropropionate ([(18) F]NFP); improved preparation of radiolabeled peptides for PET imaging. J Label Comp Radiopharm.

[24] Jin ZH, Furukawa T, Sogawa C, Claron M, Aung W, Tsuji AB (2014). PET imaging and biodistribution analysis of the effects of succinylatedgelatin combined with l-lysine on renal uptake and retention of 64Cu-cyclam-RAFT-c(-RGDfK-)4 in vivo. Eur J Pharm Biopharm.

[25] Bergeron M, Cadorette J, Tetrault M-A, Beaudoin J-F, Leroux J-D, Fontaine R (2014). Imaging performance of LabPET APD-based digital PET scanners for pre-clinical research. Phys Med Biol.

[26] Innis RB, Cunningham VJ, Delforge J, Fujita M, Gjedde A, Gunn RN (2007). Consensus nomenclature for in vivo imaging of reversibly binding radioligands. J Cereb Blood Flow Metab.

[27] Akaike H (1974). A new look at the statistical model identification. IEEE Trans Autom Control.

[28] Alves IL, VállezGarcía D, Parente A, Doorduin J, Dierckx R, Marques da Silva AM (2017). Pharmacokinetic modeling of [11C]flumazenil kinetics in the rat brain. EJNMMI Res.

[29] Bashir U, Azad G, Siddique MM, Dhillon S, Patel N, Bassett P (2017). The effects of segmentation algorithms on the measurement of 18F-FDG PET texture parameters in non-small cell lung cancer. EJNMMI Res.

[30] Matsumoto M, Hada N, Sakamaki Y, Uno A, Shiga T, Tanaka C (2013). An improved mouse model that rapidly develops fibrosis in non-alcoholic steatohepatitis. Int J Exp Pathol.

[31] Van Herck MA, Vonghia L, Francque SM (2017). Animal models of nonalcoholic fatty liver disease—a starter’s guide. Nutrients.

[32] Zhang C, Liu H, Cui Y, Li X, Zhang Z, Zhang Y (2016). Molecular magnetic resonance imaging of activated hepatic stellate cells with ultrasmallsuperparamagnetic iron oxide targeting integrin α_v_β_3_ for staging liver fibrosis in rat model. Int J Nanomed.

[33] Kleiner DE, Brunt EM, Van Natta M, Behling C, Contos MJ, Cummings OW (2005). Design and validation of a histological scoring system for nonalcoholic fatty liver disease. Hepatology.

[34] Li F, Song Z, Li Q, Wu J, Wang J, Xie C (2011). Molecular imaging of hepatic stellate cell activity by visualization of hepatic integrin α_v_β_3_ expression with SPECT in rat. Hepatology.

[35] Dobie R, Wilson-Kanamori JR, Henderson BEP, Smith JR, Matchett KP, Portman JR (2019). Single-cell transcriptomics uncovers zonation of function in the mesenchyme during liver fibrosis. Cell Rep.

[36] Wang QB, Han Y, Jiang TT, Chai WM, Chen KM, Liu BY (2011). MR Imaging of activated hepatic stellate cells in liver injured by CCl 4 of rats with integrin-targeted ultrasmallsuperparamagnetic iron oxide. Eur Radiol.

[37] Zhou X, Murphy FR, Gehdu N, Zhang J, Iredale JP, Benyon RC (2004). Engagement of αvβ3 integrin regulates proliferation and apoptosis of hepatic stellate cells. J Biol Chem.

[38] Huang XW, Wang JY, Li F, Song ZJ, Xie C, Lu WY (2010). Biochemical characterization of the binding of cyclic RGDyK to hepatic stellate cells. Biochem Pharmacol.

[39] Li D, He L, Guo H, Chen H, Shan H (2015). Targeting activated hepatic stellate cells (aHSCs) for liver fibrosis imaging. EJNMMI Res.

[40] Lukey PT, Coello C, Gunn R, Parker C, Wilson FJ, Saleem A (2019). Clinical quantification of the integrin αvβ6 by [18F]FB-A20FMDV2 positron emission tomography in healthy and fibrotic human lung (PETAL Study). Eur J Nucl Med Mol Imaging.

[41] García-Lezana T, Raurell I, Bravo M, Torres-Arauz M, Salcedo MT, Santiago A (2018). Restoration of a healthy intestinal microbiota normalizes portal hypertension in a rat model of nonalcoholicsteatohepatitis. Hepatology.

[42] Sethasine S, Jain D, Groszmann RJ, Garcia-Tsao G (2012). Quantitative histological-hemodynamic correlations in cirrhosis. Hepatology.

[43] Møller S, Henriksen JH, Bendtsen F (2014). Extrahepatic complications to cirrhosis and portal hypertension: Haemodynamic and homeostatic aspects. World J Gastroenterol.

[44] Busk TM, Bendtsen F, Henriksen JH, Fuglsang S, Clemmesen JO, Larsen FS (2017). Effects of transjugular intrahepatic portosystemic shunt (TIPS) on blood volume distribution in patients with cirrhosis. Dig Liver Dis.

[45] Van der Linden P, Le Moine O, Ghysels M, Ortinez M, Deviere J (1996). Pulmonary hypertension after transjugular intrahepatic portosystemic shunt: effects on right ventricular function. Hepatology.

[46] Møller S, Henriksen JH (2009). Cardiovascular complications of cirrhosis. Postgrad Med J..

