# Supplementary material for: Quantitative evaluation of hepatic integrin αvβ3 expression by positron emission tomography imaging using 18F-FPP-RGD2 in rats with non-alcoholic steatohepatitis
Source: EJNMMI Res. 2020 Oct 7;10:118. doi: 10.1186/s13550-020-00704-3 (PMC7541810; doi:10.1186/s13550-020-00704-3)
Supplement: Supplementary file 1 — Additional file 1. Fig. 1: TLC autoradiograms of plasma and liver extracts from rats 30 or 90 min after the intravenous administration of 18F-FPP-RGD2; Table 1: Percentages of non-metabolised 18F-FPP-RGD2 in the plasma and liver. [file 13550_2020_704_MOESM1_ESM.docx]

**Article title**

Quantitative evaluation of hepatic integrin α_v_β_3_ expression by positron-emission tomography imaging using ^18^F-FPP-RGD_2_ in rats with non-alcoholic steatohepatitis

**Journal name**

European Journal of Nuclear Medicine and Molecular Imaging Research

**Author names**

Shuichi Hiroyama, Takemi Rokugawa, Miwa Ito, Hitoshi Iimori, Ippei Morita, Hiroki Maeda, Kae Fujisawa, Keiko Matsunaga, Eku Shimosegawa, Kohji Abe

**Corresponding author**

Shuichi Hiroyama

Translational Research Unit, Biomarker R&D Department, Shionogi & Co., Ltd., Osaka, Japan

3-1-1 Futaba-cho, Toyonaka, Osaka 561-0825, Japan.

E-mail: [shuichi.hiroyama@shionogi.co.jp](mailto:shuichi.hiroyama@shionogi.co.jp)

**Additional file 1: Fig. 1** TLC autoradiograms of plasma and liver extracts from rats 30 or 90 min after the intravenous administration of ^18^F-FPP-RGD_2_

**
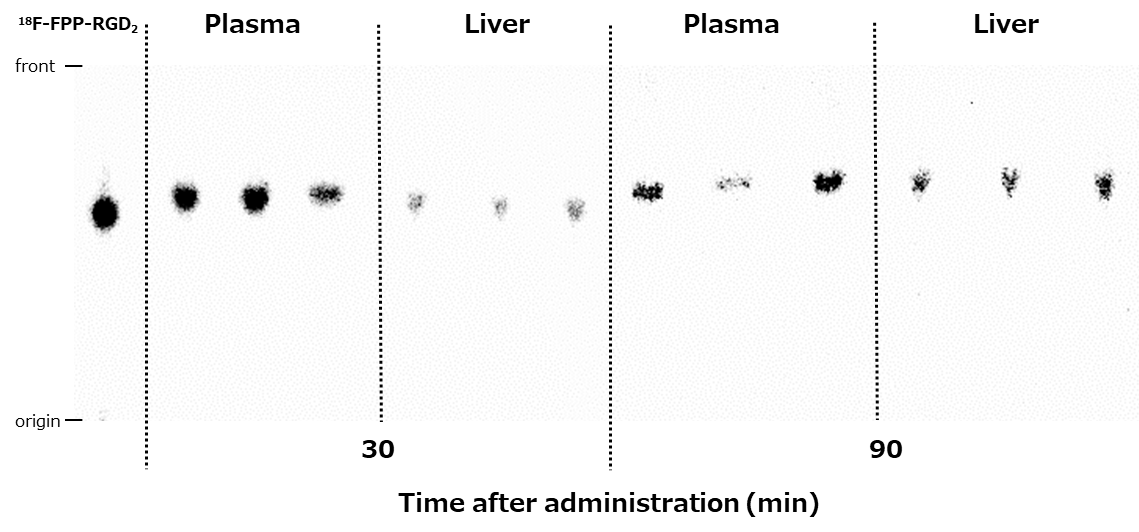
**

**Additional file 1: Table 1** Percentages of non-metabolised ^18^F-FPP-RGD_2_ in the plasma and liver

| Time (min) | Plasma |  | Liver |
| --- | --- | --- | --- |
| 30 | 99.8 ± 0.05 |  | 99.2 ± 0.17 |
| 90 | 96.9 ± 2.14 |  | 97.5 ± 0.36 |

Values are expressed as means ± SDs (n = 3).
